# Rebound Pain—Management Strategies for Transitional Analgesia: A Narrative Review

**DOI:** 10.3390/jcm14030936

**Published:** 2025-01-31

**Authors:** Kevin J. Murphy, Brian O’Donnell

**Affiliations:** Department of Anaesthesiology and Intensive Care Medicine, Cork University Hospital, T12DC4A Cork, Ireland; brian.odonnell@hse.ie

**Keywords:** peripheral nerve blocks, postoperative pain, rebound pain, regional anaesthesia, multimodal analgesia

## Abstract

Peripheral nerve blocks (PNBs), while effective in reducing postoperative opioid use and side effects, are often associated with rebound pain (RP), a significant clinical issue requiring proactive management. Methods: A systematic search of electronic databases (e.g., PubMed, EMBASE, Cochrane Library) was conducted for studies investigating rebound pain following regional anaesthesia. Recent findings: RP has a high incidence in ambulatory patients and is influenced by patient, surgical, and anaesthetic factors. Preoperative education, multimodal analgesia, continuous nerve blocks, and intravenous dexamethasone may mitigate RP. Although RP does not typically affect overall opioid use, recovery, or patient satisfaction, the majority of patients experiencing RP would still choose PNBs for future surgery.

## 1. Introduction

Several definitions of rebound pain (RP) exist in the literature. A marked increase in pain intensity upon dissipation of a peripheral nerve block (PNB) is a common feature in all RP definitions [1]. Other common defining features include pain categorised as ‘severe’ and/or causing despair following regression of PNB [2]. Rebound pain can have a profound negative impact on quality of recovery following surgery and reduced activities of daily living for a protracted period of time [1].

The aetiology of RP remains uncertain. It is unclear whether rebound pain is simply a subjective patient experience upon transiting from ‘complete regional analgesia’ to ‘no regional analgesia’; a reflection of the under-treatment of pain with appropriate, ‘round the clock dosing’ of multimodal systemic analgesia; or if RP is mechanistically related to transient nociceptive afferent blockade using sodium channel-blocking local anaesthetic agents. Some authors suggest that rebound pain and hyperalgesia share similar mechanisms [3]. Animal experiments may contribute to understanding transient hyperalgesia that can follow the resolution of a nerve block. Kolarczyk and Williams investigated behavioural changes after a single-shot ropivacaine sciatic nerve block in rats and found modest heat hyperalgesia after block resolution, without hyperalgesia to mechanical stimuli. The authors postulate the abnormal hyperactivity of C-fibers as causative [4]. Janda et al. also found evidence of transient thermal hyperalgesia in rats, approximately two hours following block resolution [5]. These experiments are of particular importance as they potentially isolate the nerve block as a principal contributing factor to RP. While bupivacaine can induce Schwann cell degeneration and demyelination, leading to transient neurotoxicity [6], this is likely not the primary cause of RP as the associated neurogenic pain is typically of longer duration [1]. Mechanical trauma from the regional anaesthesia needle insertion and local anaesthetic injectate barotrauma may also contribute to perineural inflammation [7].

In the last decade, the practice of regional anaesthesia has advanced significantly, particularly following the introduction of ultrasound-guided regional anaesthesia (UGRA). The benefits of regional anaesthesia have been well documented, and include reduced perioperative opioid use, shorter hospital stays, and the prevention of persistent post-surgical pain (PPSP) [8,9,10]. Unfortunately, rebound pain is an increasingly recognised adverse event [11]. Poorly controlled postoperative pain is associated with short-term consequences such as cardiovascular and respiratory complications, as well as long-term issues such as persistent post-surgical pain, opioid use disorder, and increased healthcare costs [12,13,14]. Although there is insufficient evidence to attribute these negative outcomes directly to RP, patient dissatisfaction and unanticipated healthcare resource utilisation have been independently associated with RP [15,16].

There is a wide variance in the incidence of rebound pain, but it is reported as being as high as 50% in patients who receive a PNB [17]. Patient risk factors associated with RP include severe pre-operative pain, female sex, and psychosocial factors such as depression [3,17]. Older patients may also experience less severe and less frequent rebound pain. A study by Sort et al. found that patients >65 years old undergoing emergency ankle fracture surgery reported lower levels of rebound pain compared to those aged between 20 and 60 years old [18]. This may be explained by age-related changes in nociceptive perception, such as a decreased sensitivity to pressure pain [19].

For procedural risk factors, patients undergoing boney surgery are 6.5 times more likely to develop rebound pain compared to those who undergo soft tissue surgery. There is a high incidence of RP in ambulatory surgery [17]. Prior to performing regional anaesthesia, the above risk factors should be considered in line with strategies for mitigation, which will be the focus of the remainder of this article.

## 2. Strategies for Mitigation

### 2.1. Patient Education

Rebound pain can occur irrespective of local anaesthetic type, volume, or concentration [20]. Thus, other factors besides the choice of local anaesthetic must be considered. Avoidance of the phenomenon of RP through prevention is the ideal objective. Patient education and management of expectations is an important factor in this. Patients expect post-surgical pain, and it is one of their most commonly reported surgical concerns [21]. It is unknown how those expectations are modified when they receive regional anaesthesia. Studies have demonstrated that individuals who expect effective pain relief often experience reduced pain perception and altered brain activity in response to painful stimuli [22]. Conversely, if these expectations are not met, it may lead to increased pain perception and reporting. This is important to consider for patients undergoing PNBs, who are often informed of significant postoperative pain relief [23]. Patients should be given a realistic expectation of block duration and understanding of the signs associated with block resolution. Educational initiatives such as pre-procedural classes and meetings with anaesthesiologists have been shown to increase the rates at which patients accept regional anaesthesia options [24,25]. Although patient education materials surrounding peripheral nerve blocks can be highly variable [26], providing written or multimedia information in addition to verbal instructions can help patients improve compliance with multimodal analgesia prior to block regression, and reduce anxiety and uncertainty before and after surgery [27].

### 2.2. Continuous PNB Catheters

The use of PNB catheters and continuous infusions of local anaesthetics might significantly reduce the occurrence of rebound pain by prolonging the effects of PNBs beyond the time point of significant nociceptive input. The prolongation of the sensory block might circumvent the abrupt end to the sensory blockade, thereby facilitating a tapering of the local anaesthetic effect [28].

Salviz et al. randomised patients undergoing arthroscopic rotator cuff repair surgery into three groups receiving general anaesthesia (GA) alone, GA combined with a single-shot interscalene block, and GA combined with a perineural interscalene catheter. The catheter group reported significantly lower pain scores than the other two groups at all time points, with a lower incidence of rebound pain [29]. Kim et al. randomised patients undergoing rotator cuff repair to receive single-shot or patient-controlled bolus analgesia via a continuous brachial plexus catheter. They report a lesser incidence in rebound pain in patients who received interscalene continuous nerve catheters at 12 h, when compared with a single-shot block [30].

Ganta and colleagues studied 50 patients who received either a single-shot infraclavicular PNB versus a continuous infusion of local anaesthetic for distal radius fractures. Although not statistically significant, the continuous infusion group had lower pain scores and had lower opioid requirements at each interval post operatively [31].

A recent study by Lee et al. found that continuous infraclavicular catheters reduced both the intensity and duration of rebound pain when compared to a single shot infraclavicular block for distal radius fixation. However, there was no difference in opioid consumption between the two groups. A potential limitation of this study is the use of intravenous dexmedetomidine for patient sedation, which may have influenced acute postoperative pain and subsequent analgesic requirements [32].

While continuous nerve blocks are effective and have become widely adopted, they are limited by certain practical considerations. Catheter migration can occur in both upper and lower extremities. Previous studies have reported catheter dislodgement rates of 21.7%, 9.1%, and 4.5% after continuous popliteal sciatic, infraclavicular, and interscalene blocks, respectively [29,32,33].

Careful patient follow-up is required to ensure both quality and safety, as delayed recovery of sensory and motor function is an inevitable consequence of continuous nerve block techniques. Previous studies have highlighted the risk of falls and pressure injuries in patients receiving continuous femoral nerve catheters [34]. Although the concentration, volume, and infusion rate of the local anesthetic may impact the preservation of motor function and proprioception, the precise relationship between these factors and clinical outcomes is uncertain and may vary depending on the specific anatomic location [35].

Perineural catheters also incur a small additional risk of block site complications [6]. The effective insertion of a PNB catheter requires a high level of expertise, and the safe use of continuous local anaesthesia infusions is both labour and material intensive. These cost and management factors have particularly hampered their use for ambulatory surgery.

### 2.3. Adjuvants

The prolongation of the effects of a PNB can be achieved through various adjuvants, which can be combined with local anaesthetic agents and administered perineurally. These include clonidine, dexmedetomidine, dexamethasone, buprenorphine, midazolam, epinephrine, tramadol, magnesium, morphine, and others [36,37]. In addition to prolonging the duration of analgesia, these adjuvants help to reduce overall dose requirements for local anaesthetics [36]. However, concerns for potential neural side effects and the toxicity of these adjuvants exist [37]. Many of these agents are not licensed for perineural use.

Dexamethasone, when given perineurally, has been shown to reduce rebound pain. Fang et al. investigated the effect of dexamethasone added to ropivacaine in patients undergoing upper limb fracture surgery. Patients who received perineural dexamethasone and ropivacaine had a five-hour mean prolongation of block, and less peak pain scores after block regression as compared to those who received ropivacaine alone [38]. It is not known how perineurally administered dexamethasone prolongs the effect of local anaesthetic agents. In mouse models of PNB, axonal degeneration and demyelination seen with the perineural administration of bupivacaine alone were significantly lessened with the admixture of perineural bupivacaine and dexamethasone [39].

Dexamethasone can also be effective in reducing RP when given intravenously. In patients undergoing ankle fracture surgery with regional anaesthesia, Gao et al. reported a reduction in rebound pain and increased PNB block duration in patients who received IV dexamethasone when compared to a control group who did not receive IV dexamethasone [40]. Interestingly, in a study by Barry et al., the absence of the intraoperative use of IV dexamethasone was cited as an independent risk factor for developing rebound pain [17]. While individual studies have indicated that perineurally administered dexamethasone may extend the duration of PNB compared to intravenous administration [41,42], a recent meta-analysis by Singh and colleagues found that both routes of administration have been shown to be equally efficacious in prolonging the duration and analgesic effect of peripheral nerve blocks. The same study also showed that the time to onset of rebound pain was also significantly delayed in the dexamethasone group compared with the control group. The optimal dose of prophylactic dexamethasone was not investigated. While the meta-analysis was limited by substantial clinical heterogeneity, the findings may be generalisable across diverse surgical populations and various nerve blocks [43].

When compared, IV and perineural dexamethasone exhibit similar safety profiles, particularly regarding postoperative nausea and vomiting and perioperative glucose levels. No long-term neurological adverse events have been associated with perineural dexamethasone, however its use remains off label [42].

The mechanism for rebound pain reduction by dexamethasone is unclear, but its role as an analgesic agent is well evidenced. It is thought to attenuate pain sensitisation by inhibiting nociceptive C-fiber transmission at the dorsal root ganglion and reducing prostaglandin production, thereby mitigating hyperalgesia [44]. As a potent anti-inflammatory agent, dexamethasone may reduce the inflammatory cascade triggered by surgical trauma and potentially modulate electrical impulses in afferent nerves supplying the injured site [45].

Ketamine offers potential to attenuate rebound pain when given perineurally. Ketamine is an NMDA antagonist which possesses anti-nociceptive and local anaesthetic properties. A recent meta-analysis of 12 RCTs examined the analgesic efficacy of perineural ketamine. The authors found that perineural ketamine was associated with a longer duration of analgesia, and lesser opioid requirement without influencing the duration of the sensory and motor block [46]. Intravenous ketamine has, however, been shown consistently to be ineffective in preventing RP. It is unclear whether perineurally administered ketamine influences RP.

Dexmedetomidine and clonidine are both centrally acting alpha-2 receptor agonists. Knight et al. found that perineurally administered dexmedetomidine, as a local anaesthetic adjuvant, appears to improve analgesia without increasing the risk of local anaesthetic neurotoxicity [36]. Dexmedetomidine is, however, associated with bradycardia and sedation when administered in this fashion [47]. This may significantly limit any potential utility. Clonidine has been shown to prolong block duration when given perineurally, without influencing the rebound pain phenomenon [47]. Both drugs carry the risk of unwanted side effects of dizziness, pruritis, headaches, and blurred vision [48].

Buprenorphine, when given perineurally, exerts its effects by blocking synaptic transmission [36]. Williams and colleagues found that buprenorphine doses exceeding 300 μg were linked to a decrease in rebound pain following PNBs for hip and knee arthroplasty, but lower doses had no such effect [47]. A further study by Tulsyan et al. found that both 150 and 300 μg doses of buprenorphine, when added to levobupivacaine for a lumbar plexus block, provided similar levels of postoperative pain relief. A dose of 300 μg, however, resulted in significant sedation and respiratory depression [49]. Hence, although buprenorphine 150 μg appears to be an optimal dose providing prolonged postoperative analgesia and minimal sedation, it has not been shown to mitigate rebound pain.

### 2.4. Multimodal Analgesia

It can be argued that a relatively abrupt unmasking of the typical nociceptive pain trajectory is purely a consequence of the inadequate pre-emptive administration of multimodal analgesia [50]. Many studies investigating rebound pain after PNB do not routinely incorporate perioperative systemic multimodal analgesia. Patients undergoing ambulatory outpatient surgery usually receive significantly less analgesic medication prior to discharge than those for whom inpatient accommodation is planned [1]. The transition of postoperative clinical care pathway oversight from anaesthesiologists to surgeons should be actively planned and managed. A collaborative multidisciplinary approach has been described by Saminiemi and colleagues as proven successful in avoiding excessive pain on block resolution [51].

Oral pain medications can start immediately after surgery, with the intent of achieving a steady state before regional anaesthesia wears off. A postoperative pain regimen for adult patients receiving a peripheral nerve block for ambulatory surgery has been proposed by Dawson et al. [52], but there are no studies to support its effectiveness in preventing RP.

Pre-emptive analgesia, where opioid medications are administered prior to block regression, has also been suggested as a means to provide a smooth transition to as-needed oral analgesics. One study demonstrated superior pain control with a lumbar plexus block compared to a fascia iliaca block for up to two hours post-arthroscopic hip surgery. Both groups received a standard dose of oxycodone postoperatively, resulting in similar opioid consumption and discharge times. The absence of postoperative rebound pain in both groups may be attributed to the preemptive analgesic effect of the opioid medication [53].

The optimal timing and dosing of postoperative analgesia is challenging due to factors such as the variable onset of rebound pain. For instance, rebound pain may occur 12–24 h after extremity fracture fixation [31,33] or 1–2 days after shoulder arthroscopy [54]. A recent study by Uppal and colleagues investigated giving 2 mg of hydromorphone six hours after an interscalene block to patients undergoing arthroscopic shoulder surgery. Although they did not find any difference in the worst pain score at 24 h compared with the placebo, it is likely that the peak effect of the study drug may not have coincided with the worst pain intensity, due to a large discrepancy between their hypothesised sensory block duration (6 h) and recruitment data (12 h) [55].

It should be noted that patients may also be reluctant to start taking pain medications, especially opioids, before experiencing pain. Additionally, these medications can have side effects like dizziness, nausea, and sedation, which can be problematic for ambulatory patients [56].

### 2.5. Pain Mechanisms

Recent advances in neuroscience and biotechnology have led to an increased understanding of the neuronal circuits and molecular mechanisms involved in pain modulation. These include genetic mutations, epigenetic and posttranslational modification, inflammasomes, signaling pathways, and microbiota [57]. This progress has facilitated the identification of novel diagnostic and therapeutic targets for different pain mechanisms [58].

Nociceptive pain, the most prevalent form of pain, originates from actual or threatened damage to non-neural tissue, leading to the activation of nociceptors. This transient response to noxious stimuli triggers protective and evasive actions. Nociplastic pain describes chronic pain conditions not attributable to direct nociceptor activation or neuropathy, but characterised by clinical and psychophysical evidence of altered nociceptive processing. Neuropathic pain is pain caused by a lesion or disease of the somatosensory nervous system. It can result from sources as varied as nerve compression, channelopathies, autoimmune disease, and incision.

Understanding the neurobiological basis of pain is key to developing mechanism-based analgesia, contingent on improved diagnostics. Elucidating neuroadaptive changes in rebound pain will enable targeted therapies. Continued translational research is essential, focusing on molecular mechanisms, targeted pharmaceutical strategies (including mechanisms of action and clinical applications), sensory phenotypes, patient clusters, and predictors of analgesic efficacy in rebound pain.

## 3. Conclusions

Peripheral nerve blocks (PNBs) have revolutionised postoperative pain management by reducing opioid exposure and associated side effects. However, PNBs are often linked to rebound pain, a common issue that requires proactive management to prevent short-term discomfort, long-term complications, and increased resource utilisation. Recent studies have shown that the incidence of this could be as high as 50% in ambulatory patients, and is influenced by patient, surgical, and anaesthetic factors [13].

Preoperative education is crucial for managing patient expectations and encouraging the early use of systemic multimodal pain management. A collaborative effort between anaesthesiologists and surgeons may optimise patient outcomes in this regard [13]. Continuous nerve block techniques can provide extended pain relief during the postoperative period, and intravenous dexamethasone may be a protective factor in preventing rebound pain. More research is needed into the use of pre-emptive analgesia as a means of mitigating against rebound pain.

Although rebound pain may occur with regional anaesthesia, no influence on cumulative postoperative opioid use, patient recovery outcomes, and patient satisfaction has been identified. Interestingly, despite the occurrence of rebound pain, 96% of patients expressed a preference for nerve blocks in future surgical procedures [17].

Rebound pain is a problem that requires active management to mitigate negative effects on the patient experience.
